# Supplementary material for: The intergenerational transmission of educational attainment: A closer look at the (interrelated) roles of paternal involvement and genetic inheritance
Source: PLoS One. 2022 Dec 12;17(12):e0267254. doi: 10.1371/journal.pone.0267254 (PMC9744317; doi:10.1371/journal.pone.0267254)
Supplement: S1 File — (DOCX) [file pone.0267254.s001.docx]

**Supporting information, tables and figures**

**S1 Table** Results from the multilevel path model on years of education child

**S2 Table** Effect of the Education PGS on years of education in the 1) between family model with only genetic PCs as controls, 2) between family model including genetic PCs and additional controls and 3) Within family sibling model

**S1 Robustness checks regarding the education PGS**

**S3 Table** Effect of the Education PGS on years of education

**S1 Fig.** Association between education PGS and years of schooling in the complete sample

**S2 Fig.** Association between the difference in education PGS and difference in years of education between siblings.

**S2 Robustness checks regarding father involvement**

**S4 Table** Correlation between age of the respondent at the first wave, and measures of father involvement

**S5** **Table** Mean and SD of measures of father involvement for the different ages

**S3 Fig. Graphical presentation of the mediation and confounding results**

**S3 Education of parent reported by the respondent and by the parent**

**Table S1** Results from the multilevel path model on years of education child

|  | Model 1 | |  | Model 2 | | | Model 3 | |  |
| --- | --- | --- | --- | --- | --- | --- | --- | --- | --- |
|  | *β* | SE | p-value | *β* | SE | p-value | *β* | SE | p-value |
| Outcome years of education child |  |  |  |  |  |  |  |  |  |
| Father’s school-specific involvement | 0.056 | 0.016 | 0.001 |  |  |  | 0.050 | 0.016 | 0.001 |
| Father’s leisure involvement | 0.030 | 0.014 | 0.033 |  |  |  | 0.031 | 0.014 | 0.023 |
| PGS education child |  |  |  | 0.220 | 0.013 | 0.000 | 0.219 | 0.013 | 0.000 |
| Years of education father | 0.267 | 0.015 | 0.000 | 0.235 | 0.015 | 0.000 | 0.226 | 0.015 | 0.000 |
| Years of education mother | 0.235 | 0.015 | 0.000 | 0.195 | 0.015 | 0.153 | 0.197 | 0.015 | 0.000 |
| Age w1 | 0.029 | 0.013 | 0.060 | 0.022 | 0.012 | 0.098 | 0.027 | 0.012 | 0.048 |
| Sex = male | -0.253 | 0.026 | 0.000 | -0.254 | 0.025 | 0.000 | -0.266 | 0.025 | 0.000 |
| Father = resident | 0.228 | 0.029 | 0.000 | 0.208 | 0.028 | 0.000 | 0.212 | 0.028 | 0.000 |
| Enrolled = Y | 0.191 | 0.046 | 0.000 | 0.176 | 0.044 | 0.000 | 0.172 | 0.044 | 0.000 |
| Mother = resident | 0.294 | 0.045 | 0.000 | 0.261 | 0.044 | 0.000 | 0.270 | 0.044 | 0.000 |
| Mother’s school-specific involvement | 0.050 | 0.016 | 0.002 | 0.073 | 0.013 | 0.000 | 0.043 | 0.015 | 0.005 |
| Mother’s leisure involvement | 0.041 | 0.014 | 0.004 | 0.050 | 0.013 | 0.000 | 0.038 | 0.014 | 0.010 |
| R2 | 0.372 |  |  | 0.405 |  |  | 0.404 |  |  |

| **Table S1** *Continued* |  |  |  |  |  |  |  |  |  |
| --- | --- | --- | --- | --- | --- | --- | --- | --- | --- |
|  | Model 1 | |  | Model 2 | |  | Model 3 |  |  |
|  | β | SE | p-value | β | SE | p-value | β | SE | p-value |
| Outcome father-child school contact |  |  |  |  |  |  |  |  |  |
| Years of education father | 0.144 | 0.015 | 0.000 |  |  |  | 0.144 | 0.015 | 0.000 |
| R2 | 0.021 |  |  |  |  |  | 0.021 |  |  |
| Outcome father-child activities |  |  |  |  |  |  |  |  |  |
| Years of education father | 0.142 | 0.015 | 0.000 |  |  |  | 0.142 | 0.015 | 0.000 |
| R2 | 0.020 |  |  |  |  |  | 0.020 |  |  |
| Outcome PGS education child |  |  |  |  |  |  |  |  |  |
| Years of education father |  |  |  | 0.299 | 0.014 | 0.000 | 0.299 | 0.014 | 0.000 |
| R2 |  |  |  | 0.112 |  |  | 0.112 |  |  |
| Total and indirect effect of education father |  |  |  |  |  |  |  |  |  |
| Indirect via father’s school-specific involvement | 0.008 | 0.002 | 0.002 |  |  |  | 0.007 | 0.002 | 0.005 |
| Indirect via father’s leisure involvement | 0.004 | 0.002 | 0.037 |  |  |  | 0.004 | 0.002 | 0.027 |
| Indirect via PGS |  |  |  | 0.066 | 0.005 | 0.000 | 0.065 | 0.005 | 0.000 |
| Direct effect | 0.267 | 0.015 | 0.000 | 0.235 | 0.015 | 0.000 | 0.226 | 0.015 | 0.000 |
| Total effect | 0.279 | 0.016 | 0.000 | 0.300 | 0.015 | 0.000 | 0.303 | 0.016 | 0.000 |
| N individuals | 4579 |  |  | 4579 |  |  | 4579 |  |  |
| N Household | 4154 |  |  | 4154 |  |  | 4154 |  |  |

*Note: In our path to educational attainment and to education PGS we control for the first 10 principal components. All measures are standardized.*

**Table S2** Correlation table

|  | educ | educ father | Educ mother | pgs | father school | father activities | mother school | mother activities | age w1 | age final | father res | mother resi |
| --- | --- | --- | --- | --- | --- | --- | --- | --- | --- | --- | --- | --- |
| education father | 0.45 |  |  |  |  |  |  |  |  |  |  |  |
|  | (0.00) |  |  |  |  |  |  |  |  |  |  |  |
| education mother | 0.43 | 0.56 |  |  |  |  |  |  |  |  |  |  |
|  | (0.00) | (0.00) |  |  |  |  |  |  |  |  |  |  |
| pgs | 0.38 | 0.31 | 0.30 |  |  |  |  |  |  |  |  |  |
|  | (0.00) | (0.00) | (0.00) |  |  |  |  |  |  |  |  |  |
| father school contact | 0.17 | 0.14 | 0.10 | 0.09 |  |  |  |  |  |  |  |  |
|  | (0.00) | (0.00) | (0.00) | (0.00) |  |  |  |  |  |  |  |  |
| father activities | 0.12 | 0.14 | 0.11 | 0.06 | 0.30 |  |  |  |  |  |  |  |
|  | (0.00) | (0.00) | (0.00) | (0.00) | (0.00) |  |  |  |  |  |  |  |
| mother school contact | 0.16 | 0.10 | 0.12 | 0.09 | 0.60 | 0.12 |  |  |  |  |  |  |
|  | (0.00) | (0.00) | (0.00) | (0.00) | (0.00) | (0.00) |  |  |  |  |  |  |
| mother activities | 0.16 | 0.06 | 0.12 | 0.05 | 0.17 | 0.33 | 0.28 |  |  |  |  |  |
|  | (0.00) | (0.00) | (0.00) | (0.00) | (0.00) | (0.00) | (0.00) |  |  |  |  |  |
| age w1 | -0.03 | -0.04 | -0.05 | -0.01 | -0.07 | -0.06 | -0.06 | 0.00 |  |  |  |  |
|  | (0.05) | (0.00) | (0.00) | (0.45) | (0.00) | (0.00) | (0.00) | (0.82) |  |  |  |  |
| age final | 0.20 | 0.09 | 0.06 | 0.07 | -0.01 | -0.05 | 0.00 | 0.01 | 0.35 |  |  |  |
|  | (0.00) | (0.00) | (0.00) | (0.00) | (0.70) | (0.00) | (0.97) | (0.53) | (0.00) |  |  |  |
| father resident | 0.14 | 0.13 | 0.06 | 0.06 | -0.02 | -0.04 | -0.04 | -0.09 | -0.03 | 0.05 |  |  |
|  | (0.00) | (0.00) | (0.00) | (0.00) | (0.13) | (0.01) | (0.02) | (0.00) | (0.02) | (0.00) |  |  |
| mother resident | 0.14 | 0.07 | 0.12 | 0.07 | 0.02 | -0.03 | 0.03 | 0.07 | -0.13 | 0.00 | 0.04 |  |
|  | (0.00) | (0.00) | (0.00) | (0.00) | (0.13) | (0.03 | (0.05) | (0.00) | (0.00) | (0.99) | (0.01) |  |
| enrolled w1 | 0.06 | 0.00 | 0.00 | 0.02 | 0.02 | 0.03 | 0.03 | 0.04 | -0.03 | -0.10 | 0.00 | 0.00 |
|  | (0.00) | (0.84) | (0.81) | (0.11) | (0.17) | (0.02) | (0.09) | (0.02) | (0.05) | (0.00) | (0.76) | (0.99) |

*Note: Education=Years of education child; Education father=Years of education father; Education mother=Years of education mother; pgs=Child’s Polygenic score for education; father school contact=father’s school-specific involvement; father activities=father’s leisure involvement; mother school contact=Mother’s school-specific involvement; mother activities=mother’s leisure involvement; age w1= age at wave 1; age final=age at the final wave; father resident= lived with father at wave 1, 0= no 1=yes; mother resident= lived with mother at wave 1, 0= no 1=yes; enrolled w1: was the respondent enrolled in education at wave 1, 0=no, 1=yes. P-values are displayed between parentheses.*

**S1 Robustness checks regarding the education PGS**

Inflation of PGS due to indirect effects

The effect of the education PGS can be “directly” related to education or more indirectly via the family environment. In our main analyses, we show that this is partly through one specific aspect of the family environment, namely father involvement. Within family sibling models control for all measures and unmeasured family and environmental factors that vary across families. This gives a more complete picture of possible social genetic effects. As can be seen in Table S3 as well as Figure A1 and A2, the education PGS is significantly related to years of education in the between family as well as in the within family model. However, the estimates in the between family model are smaller. The estimate in the within family model is 0.142, while the estimate in the between family model with only the first 10 genetic PCs as control variables is 0.366 and in the model with additional controls it is 0.219.

This smaller effect in the within family model is in line with previous research that shows that the effect of SNPs within families is roughly 40% to 60% smaller than between families (1–3). This is likely due to confounding by the rearing environment (4) and is partly explained by family SES (3). This smaller effect of the education PGS within families is also observed for previous versions of the PGS (5). These findings are however contrary to the findings of Conley and colleagues, who found stronger effects of the educational PGS on education within families, which they argue could be due to niche formation or social distinction, in which small differences between siblings might cause parents to behave differently to their children, which might reinforce initial differences (6). Also in the Add Health dataset, Domingue and colleagues found that within siblings the effect of the PGS on education is just as large as between families (7).

**Table S3** Effect of the Education PGS on years of education in the 1) Between family model with only genetic PCs as controls, 2) Between family model including genetic PCs and additional controls and 3) Within family sibling model

|  | B | SE | significance | N |
| --- | --- | --- | --- | --- |
| Between family PC | 0.366 | 0.013 | *** | 4579 |
| Between family PC + controls | 0.219 | 0.013 | *** | 4579 |
| Within family | 0.142 | 0.47 | ** | 380 |

*Note: +p<0.1, *p<0.05, **p<0.01, ***p<0.001. The first OLS model controls for* *the first 10 genetic PCs, The second OLS model controls for the first 10 genetic PCs, age first interview, sex, enrolled in the last wave, father involvement, mother involvement, whether child lived with father and mother, and the educational attainment of the parents. Sample size in the within family models refers to the nr of sibling pairs.*


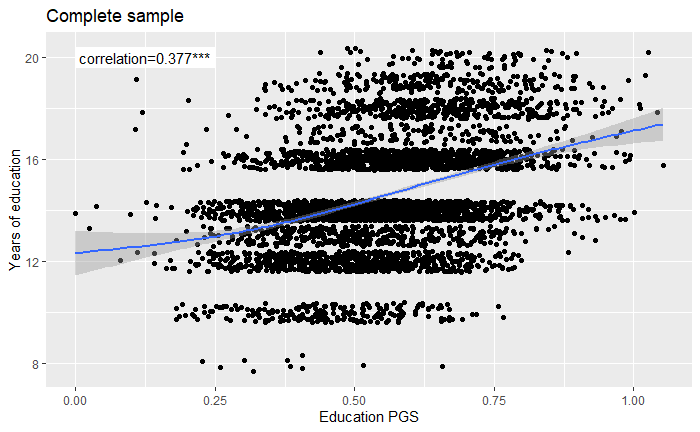


**Figure S1** Association between education PGS and years of schooling in the complete sample


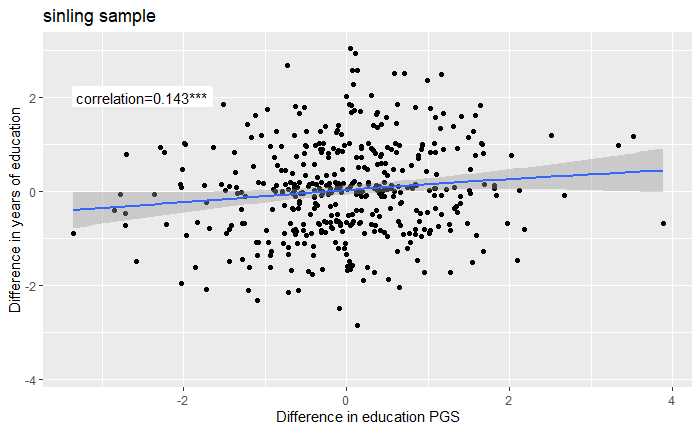


**Figure S2** Association between the difference in education PGS and difference in years of education between siblings.

Inflation of genetic effects due to population stratification.

The genotype-phenotype association might be overestimated due to population stratification. To control for this, we make use of a homogeneous sample of only European Ancestry individuals and control for the first 10 principal components. Yet, the population stratification might still not be fully controlled for by the first 10 genetic PCs (8). To test if there is population stratification inflating results, we can estimate if the association PGS and education reduces when we include the first 10 genetic PCs.

We find that there is a slight reduction when we include the 10 genetic PCs. Without the coefficient is 0.376 (SE 0.013) and with it is 0.366 (SE 0.013) (reduction of 2.65%). This indicates that population stratification might to a small extent be causing an overestimation of our results.

A more stringent test to control for population stratification is to use a sibling design and examine if differences between siblings’ PGS relate to differences in siblings’ years of education (8). In this model, the coefficient of the PGS is 0.142 (SE 0.047) (see Table S2 and Figure S1 and S2). However, as described above this estimate does not only control for population stratification, but also for indirect genetic effects (which is one of the main interests of our study).

Genetic assortative mating on education impacting the results of the PGS

Previous research has found that partners are more similar to each other concerning education and education-related genes than random individuals (9). Estimates from our study also show that the correlation between years of education of the parents is 0.55, which is in line with findings from previous studies (8). Previous research furthermore found significant correlations between the PGS of partners of 0.18 (8), 0.11 (10). This positive genetic correlation between parents could also cause siblings to be more similar to each other on education-related genes, than the usual average genetic similarity of 0.50 between siblings. In our sample, the similarity in the PGS between siblings is 0.52 (CI between 0.448 and 0.587), which is slightly higher yet not significantly higher than the expected 0.50. This deviation from 0.5 is possibly due to the assortative mating of the parents.

Assortative mating of parents could in turn result in biased PGS phenotype associations (8,11). The reason is that assortative mating will result in a correlation between physically distant causal loci (long-range linkage or gametic phase disequilibrium) (11). Within GWAS studies, long-range linkage will result in overestimation of SNP effects, as they do not only capture the effect of a causal SNP but also of a correlated other causal and distal SNP (for example on another chromosome). In turn, PGS created from these GWASs will double count the effect of causal variants. Furthermore, the indirect effects of the education-related genes, that operate through parenting, results in an overestimation of the PGS effect (the alleles that children receive from their father do not only relate to the parenting of the father, but also to the parenting of the mother, and vice versa). Our findings of a slightly higher than expected sibling correlation show that the education PGS might to a small extent be an overestimation.

**S2 Robustness checks regarding father involvement**

Age of information on father involvement

Some children were already 18 years old at the first wave while others were 14 years old. At age 14, some aspects of father involvement, such as help with schoolwork was probably more common and more relevant. Therefore, we estimated the correlation between the different aspects of father involvement and the age at which the respondent reported about this involvement. We found that all aspects of father involvement were lower among older respondents compared to younger respondents (see Table A3). Furthermore, respondents who were older at the first wave also obtained slightly less years of education. Therefore, it is justified that we include age at the first wave as a control variable in our model.

**Table S4** Correlation between age of the respondent at the first wave, and measures of father involvement

|  | Age of respondent |
| --- | --- |
| Years of education | -0.032* |
| Father’s school-specific involvement | -0.072*** |
| Father’s leisure involvement | -0.059*** |

However, it is also possible that the effect of father involvement differs at different ages of the child. Regarding the variation of father involvement at different ages, we expect that variation is larger at younger ages, as help with schoolwork is less common at older ages. Therefore, we also expect that the relevance of father involvement is greater at younger ages. We find that the variation is indeed lower for father’s school-specific involvement at older ages, while the variation in father’s leisure involvement remains somewhat stable across ages (see Table A4).

Given that father’s school-specific involvement and variation in father’s school-specific involvement was higher at younger ages, and because theoretically, this might also be a more important predictor of school achievement at younger ages, we run regression models in which we include an interaction between father involvement and age of the respondent. We did not find a significant interaction between age at first interview and father’s school-specific involvement (b=0.053, SE=0.031, p=0.086). Given that this interaction is not significant, we can conclude that controlling for age at first measurement would be sufficient and we do not need to include interactions in our main models.

**Table S5** Mean and SD of measures of father involvement for the different ages

| Age | Father’s school-specific involvement | | Father’s leisure involvement | |  |
| --- | --- | --- | --- | --- | --- |
|  | Mean | SD | Mean | SD | |
| 13 | 1.25 | 1.08 | 1.53 | 1.25 | |
| 14 | 1.18 | 1.04 | 1.49 | 1.28 | |
| 15 | 1.20 | 1.01 | 1.52 | 1.31 | |
| 16 | 1.22 | 0.98 | 1.48 | 1.32 | |
| 17 | 1.17 | 0.96 | 1.38 | 1.27 | |
| 18 | 1.07 | 0.97 | 1.35 | 1.24 | |
| 19 | 0.89 | 0.93 | 1.24 | 1.33 | |

Father involvement and mother involvement

The correlation between the involvement of the mother and involvement of the father is 0.60 for school-specific involvement and 0.33 for leisure involvement , all significant (p<0.001). To assess whether father involvement and mother involvement could be added simultaneously to the model, we estimated the variance inflation factor, which was between 1.24 and 1.82, which is below the critical VIF value of 2.5 (12). This indicates that we do not encounter any multicollinearity issues and that, therefore, father and mother involvement can be simultaneously included in our models. (The VIF is calculated by running regression models for each aspect of father involvement, in which we predict each aspect of father involvement by all other variables in our model, namely the aspects of mother involvement, the other aspects of father involvement, our control variables (age of the child, sex of the child, enrolled in the first wave and the years of education of the parents). The VIF is calculated by 1/(1-r^2^). If the r^2^ of a model is very high, this results in a high VIF, and indicates that father involvement can be very well predicted by the other variables in our model.)

Differences between the complete sample and a sample with only resident fathers

We examined whether and to what extent our findings regarding father involvement and genetic and social confounding differed between our complete sample and our sample with resident fathers only. The results of the sample with resident fathers are displayed in Figure S3. We find that the results are largely comparable, which suggests that our findings are not driven by differences in father involvement and educational outcomes between children with resident versus non-resident fathers.


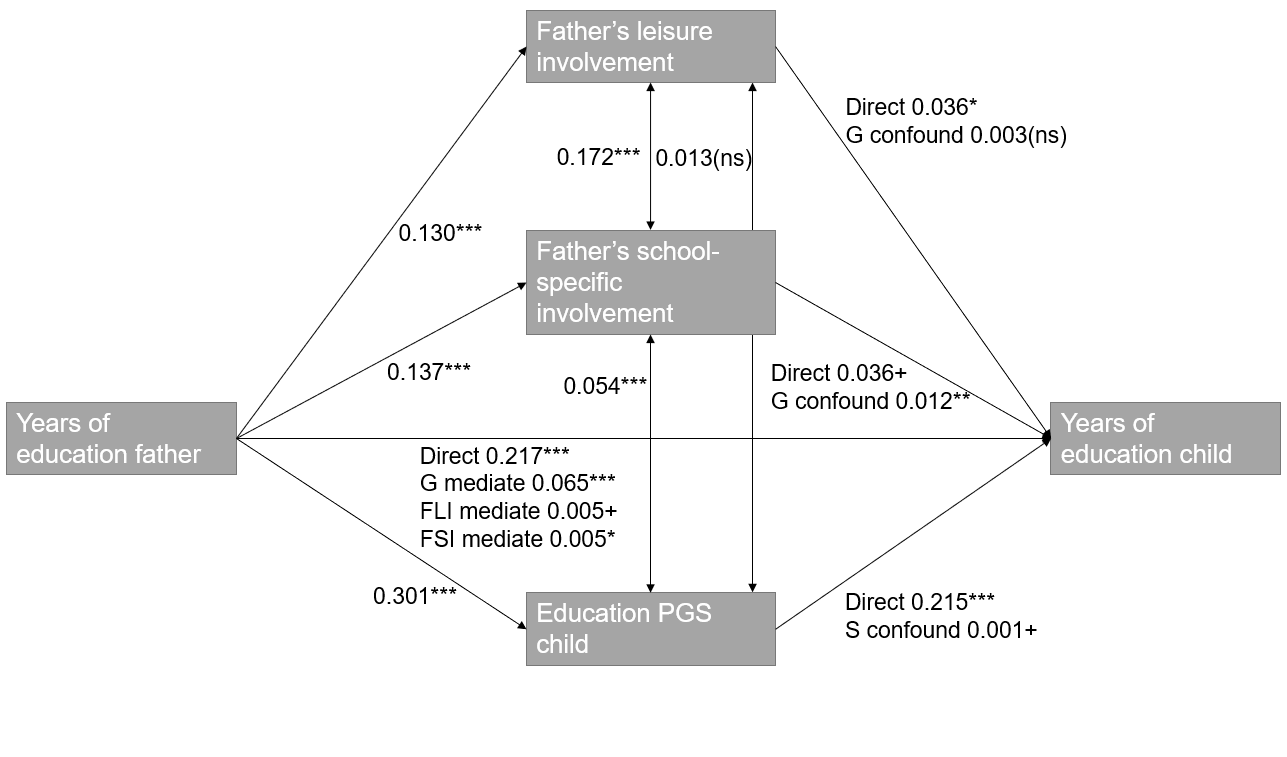


**Fig S3. Graphical presentation of the mediation and confounding results.** *G mediate refers to the part of the effect that is genetically mediated, FLI mediate refers to the mediated effect by father’s leisure involvement, FSI mediate refers to the mediated effect by father’s school-specific involvement, G confound to the genetically confounded part and S confound to the social confounding by father involvement.*

**S3 Education of parent reported by the respondent and by the parent**

In total 4072 respondents had a mother who filled in the parent questionnaire, and 189 had a father who filled in the parent questionnaire. For those fathers and mothers, we have a measure of their educational attainment reported by themselves and by their children. For mothers, the correlation between these two measures is 0.874 (p<0.001) and for fathers, this correlation is 0.790 (p<0.000).

The biggest error lies in parents who indicated that they went to college but did not graduate (coded into 14 years of education), as their children in 23% of the cases reported that their parent finished high school only (coded into 12 years of education) and 14% indicated that their parent finished a Bachelor (coded into 16 years of education) (and 60% correctly indicated the educational attainment of their parent).

**References for Supporting information**

1. Lee JJ, Wedow R, Okbay A. Gene discovery and polygenic prediction from a genome-wide association study of educational attainment in 1.1 million individuals. Nat Genet. 2018;50:1112–21.

2. Howe LJ, Chittoor G, Lind PA, Nivard MG, Morris TT, Wang Y, et al. Within-sibship GWAS improve estimates of direct genetic effects. bioRxiv. 2021;

3. Selzam S, Ritchie SJ, Pingault JB, Reynolds CA, O’Reilly PF, Plomin R. Comparing Within- and Between-Family Polygenic Score Prediction. Am J Hum Genet. 2019;105(2):351–63.

4. Lee JJ, Wedow R, Okbay A. Supplementary Note for Gene discovery and polygenic prediction from a 1 . 1-million-person GWAS. Nat Genet. 2018;50:1–205.

5. Okbay A, Beauchamp JP, Fontana MA, Lee JJ, Pers TH, Rietveld CA, et al. Genome-wide association study identifies 74 loci associated with educational attainment. Nature [Internet]. 2016;533(7604):539–42. Available from: http://www.nature.com/doifinder/10.1038/nature17671

6. Conley D, Domingue BW, Cesarini D, Dawes C, Rietveld CA, Boardman JD. Is the effect of parental education on offspring biased or moderated by genotype? Sociol Sci. 2015 Feb 25;2:82–105.

7. Domingue BW, Belsky DW, Conley D, Mullan Harris K, Boardman JD. Polygenic Influence on Educational Attainment: New evidence from The National Longitudinal Study of Adolescent to Adult Health. AERA Open. 2015;1(3):1–13.

8. Morris TT, Davies NM, Hemani G, Smith GD. Population phenomena inflate genetic associations of complex social traits. Sci Adv. 2020;6(16).

9. Robinson MR, Kleinman A, Graff M, Vinkhuyzen AAE, Couper D, Miller MB, et al. Genetic evidence of assortative mating in humans. Nat Hum Behav [Internet]. 2017;1:1–13. Available from: http://www.nature.com/articles/s41562-016-0016

10. Hugh-Jones D, Verweij KJH, St. Pourcain B, Abdellaoui A. Assortative mating on educational attainment leads to genetic spousal resemblance for polygenic scores. Intelligence [Internet]. 2016;59:103–8. Available from: http://dx.doi.org/10.1016/j.intell.2016.08.005

11. Yengo L, Robinson MR, Keller MC, Kemper KE, Yang Y, Trzaskowski M, et al. Imprint of assortative mating on the human genome. Nat Hum Behav [Internet]. 2018;2(12):948–54. Available from: http://dx.doi.org/10.1038/s41562-018-0476-3

12. Thompson CG, Kim RS, Aloe AM, Becker BJ. Extracting the variance inflation factor and other multicollinearity diagnostics from typical regression results. Basic Appl Soc Psych [Internet]. 2017;39(2):81–90. Available from: http://dx.doi.org/10.1080/01973533.2016.1277529
