# Supplementary material for: The intergenerational transmission of educational attainment: A closer look at the (interrelated) roles of paternal involvement and genetic inheritance
Source: PLoS One. 2022 Dec 12;17(12):e0267254. doi: 10.1371/journal.pone.0267254 (PMC9744317; doi:10.1371/journal.pone.0267254)
Supplement: Attachment — Submitted filename: Response to reviewers.pdf [file pone.0267254.s002.pdf]

Response to reviewers

We thank the editor and the reviewers for their very helpful suggestions to improve our paper. We have done our utmost best to carefully address all the reviewers' comments. Our replies and descriptions of the changes we made are provided in red in this response letter. Additions made to the manuscript are indicated in this letter by *italics*. Removed text is indicated by track changes. In the manuscript itself, alterations are marked solely with 'track changes'. Please note that page and line numbers that we mention in this revision memo refer to our revised manuscript (the version that visibly includes track changes).

Journal Requirements:

When submitting your revision, we need you to address these additional requirements.

1. Please ensure that your manuscript meets PLOS ONE's style requirements, including those for file naming. The PLOS ONE style templates can be found at https://journals.plos.org/plosone/s/file?id=wjVg/PLOSOne_formatting_sample_main_body.pdf and https://journals.plos.org/plosone/s/file?id=ba62/PLOSOne_formatting_sample_title_authors_affiliations.pdf

Response: In revising our manuscript, we have done our utmost best to ensure that our manuscript meets PLOS ONE's style requirements.

2. Thank you for stating the following in the Acknowledgments Section of your manuscript:
"The present study was supported by a grant from the Netherlands Organization for Scientific Research to RK (NWO MaGW VIDI; grant no. 452-17-005) and by a grant from the European Research Council to RK (ERC StG; grant no. 757210). Both authors declare no conflict of interest.
Add Health is directed by Robert A. Hummer and funded by the National Institute on Aging cooperative agreements U01 AG071448 (Hummer) and U01AG071450 (Aiello and Hummer) at the University of North Carolina at Chapel Hill. Waves I-V data are from the Add Health Program Project, grant P01 HD31921 (Harris) from Eunice Kennedy Shriver National Institute of Child Health and Human Development (NICHD), with cooperative funding from 23 other federal agencies and foundations. Add Health was designed by J. Richard Udry, Peter S. Bearman, and Kathleen Mullan Harris at the University of North Carolina at Chapel Hill."
We note that you have provided funding information that is not currently declared in your Funding Statement. However, funding information should not appear in the Acknowledgments section or other areas of your manuscript. We will only publish funding information present in the Funding Statement section of the online submission form.
Please remove any funding-related text from the manuscript and let us know how you would like to update your Funding Statement. Currently, your Funding Statement reads as follows:
"The present study was supported by a grant from the Netherlands Organization for Scientific Research to RK (NWO MaGW VIDI; grant no. 452-17-005) (https://www.nwo.nl/en) and by a grant from the European Research Council to RK (ERC StG; grant no. 757210) ()."
Please include your amended statements within your cover letter; we will change the online submission form on your behalf.

Response: We removed funding information from our acknowledgements section. In addition to what is now placed in the Funding section, we would like to kindly ask the editor if they could add the following information:

"Add Health is directed by Robert A. Hummer and funded by the National Institute on Aging cooperative agreements U01 AG071448 (Hummer) and U01AG071450 (Aiello and Hummer) at the University of North Carolina at Chapel Hill. Waves I-V data are from the Add Health Program Project, grant P01 HD31921 (Harris) from Eunice Kennedy Shriver National Institute of Child Health and Human Development (NICHD), with cooperative funding from 23 other federal agencies and foundations".

Reviewers' comments:

Reviewer's Responses to Questions

Comments to the Author

1. Is the manuscript technically sound, and do the data support the conclusions?

The manuscript must describe a technically sound piece of scientific research with data that supports the conclusions. Experiments must have been conducted rigorously, with appropriate controls, replication, and sample sizes. The conclusions must be drawn appropriately based on the data presented.

Reviewer #1: Yes
Reviewer #2: Partly
Reviewer #3: Yes
Reviewer #4: Yes

---

2. Has the statistical analysis been performed appropriately and rigorously?

Reviewer #1: Yes
Reviewer #2: I Don't Know
Reviewer #3: Yes
Reviewer #4: Yes

---

3. Have the authors made all data underlying the findings in their manuscript fully available?

The requires authors to make all data underlying the findings described in their manuscript fully available without restriction, with rare exception (please refer to the Data Availability Statement in the manuscript PDF file). The data should be provided as part of the manuscript or its supporting information, or deposited to a public repository. For example, in addition to summary statistics, the data points behind means, medians and variance measures should be available. If there are restrictions on publicly sharing data—e.g. participant privacy or use of data from a third party—those must be specified.

Reviewer #1: No
Reviewer #2: No
Reviewer #3: No
Reviewer #4: Yes

Response: Based on this information, we have now added information about our data restrictions in our data availability statement.

---

4. Is the manuscript presented in an intelligible fashion and written in standard English?

PLOS ONE does not copyedit accepted manuscripts, so the language in submitted articles must be clear, correct, and unambiguous. Any typographical or grammatical errors should be corrected at revision, so please note any specific errors here.
Reviewer #1: Yes
Reviewer #2: Yes
Reviewer #3: Yes
Reviewer #4: Yes

---

5. Review Comments to the Author

Please use the space provided to explain your answers to the questions above. You may also include additional comments for the author, including concerns about dual publication, research ethics, or publication ethics. (Please upload your review as an attachment if it exceeds 20,000 characters)

Reviewer #1: Thank you for inviting me to review this manuscript, which analyses the contributions of genes and paternal involvement to the intergenerational transmission of educational attainment. Data come from the Add Health Study of 5,021 genotyped adolescents and their parents. The study finds that both genes and father involvement (over and above mother involvement) are contribute to the intergenerational transmission of educational attainment; that these mechanisms are correlated with each other; and that part of the association of fathers' school- specific involvement and educational attainment is potentially confounded by children's education genetics.

Overall I thought this study was well-done and well-written. I particularly appreciated how the manuscript very clearly and systematically laid out and tested each hypothesis. I only have a few comments; most of these are about the language used when describing how different variables relate to each other. The study cannot establish causality, yet the language that is used often implies causality. This is problematic especially in genetics studies, because genetics studies are very prone to misinterpretation (which can have real-life devastating consequences, see recent events in the US). I used the following guide to replace terms in the paper, I may not have found every instance of the words used though and would ask the authors to carefully go through their manuscript and replace causal language with the appropriate wording:

shaping = is associated with
results in = is associated with
has an effect on = is associated with
impacts on = is associated with
leads to = is associated with

My list of suggested word-changes with page number is appended below; other comments I had were the following:

Response: We thank the reviewer for their positive evaluation of our manuscript and their constructive comments. We highly appreciate the detailed suggestions provided by the reviewer to replace causal language. Throughout the manuscript, we have changed our wording accordingly and we double-checked our manuscript for causal language before resubmitting it.

(1) For a general readership, it would be helpful to explain a bit more what a polygenic score is (i.e. explain what a GWAS does; emphasise that this is an aggregate score made up of many genetic variants, i.e. it is not a candidate gene study.

Response: Based on the reviewer's comment, we have now elaborated on the explanation of what a polygenic risk score is at the end of the introduction. This text reads as follows (p.6): *We use children's PGS for educational attainment as our genetic indicator. This PGS is based on a genome-wide association study (GWAS) conducted among 1.1 million individuals (26). In this GWAS, the association between hundreds of thousands of genetic variants and educational attainments is assessed. These GWAS summary statistics are used to calculate the sum of all risk alleles, weighted by their reported effect sizes. A PGS can therefore be seen as the summary measure of the genetic propensity for a trait based on a large number of genetic variants (27). The Education PGS has been found to explain about 11-13% of the variation in educational attainment (26).*

(2) On Page 4 and 6 it says in the headlines for each hypothesis "independent" (e.g. "Genetic influences as an independent mechanism underlying the intergenerational transmission of educational attainment") – it is not clear what the "independent" refers to (independent of what?) - would delete or clarify

Response: With 'independent' we meant to convey the association that remains to exist when we also control for other characteristics (such as mother's involvement and genetic information). Re-reading our manuscript, we understand that this doesn't come across as clearly as we had hoped. Based on the reviewer's suggestion, we have decided to remove the word 'independent' from the headlines of each hypothesis.

(3) On Page 7 it says "children inherit half of their genes from each parent, and these parents also rear them and shape their environment." it would be good to emphasise that this is only the case in biological families, which not all families are (e.g. sth like "children inherit half of their genes from each biological parent, and, if children live with their biological parents, these same parents also rear them and shape their environment)

Response: We agree with the reviewer's comment, and we have revised our manuscript in line with the reviewer's suggestion. The text now reads as follows (p.10): *children inherit half of their genes from each biological parent, and, if children live with their biological parents, these same parents also rear them and shape their environment*.

(4) The distinction between direct and indirect effects (p9) is a little vague. Direct effects are genetic associations with an individual's outcome that originate in that individual's genetics; indirect genetic effects are associations that originate in another individual's genetics (e.g. parents). In the current description it sounds as if direct genetic effects refer to effects that are mediated by a person's behaviour, and indirect genetic effects are effects that are mediated by environments. However, this is incorrect, because effects of an individual's genetics could be mediated by the environment (e.g. via evocative gene-environment correlation) yet they would still be direct effects (because they originate in that individual's genetics). See also this paper:

Young, A. I., Benonisdottir, S., Przeworski, M., & Kong, A. (2019). Deconstructing the sources of genotype-phenotype associations in humans. Science, 365(6460), 1396-1400.

Response: We thank the reviewer for this comment. We agree with the reviewer that effects of father involvement that are evoked by the genes of the child should indeed be labeled as direct genetic effects. To avoid confusion, we removed the labels direct and indirect effects in this paragraph, and rewrote this section as following (p.12):
Yet, these associations do not imply causation, and the pathways from genetic variants to education are diverse (Young et al, 2019). GWAS studies that are used to create PGSs cannot distinguish between associations between genes and education through personal traits, such as intelligence and motivation, and associations between genes and education due to the environment, such as the family environment and parenting practices.

(5) The methods say "Non-European descent individuals were removed from the sample" – how was this done? Using self-identified ancestry? Genetically-identified ancestry?

Response: The removal of individuals with non-European descents was done using generically determined ancestry. We now added this information briefly in the manuscript and refer to the quality control report by Highland (2018), in which this is further explained.

(6) What are the estimates in Table 2 – standardised beta coefficients? Or unstandardised estimates? Please clarify.

Response: These coefficients are standardized estimates, predicting the change in a standardized measure of father involvement by a standardized measure of the PGS. We now clarify this in the note below our table, as well as under figure 3 and Table S1.

(7) Authors interpret their findings to suggest that "that a substantial part of the role the father's school-specific involvement plays in the intergenerational transmission of years of education is genetically confounded" (p 21), but the confounding is 10%. That doesn't seem very substantial. Would rephrase accordingly.

Response: We agree with the reviewer that we should have toned down our language here. Based on the reviewer's comment, we have changed our wording to '*a small, but nonnegligible, part*'.

(8) Another proposed robustness check: what happens, at least for the main analyses (as the sibling sample would probably end up too small), if the sample is restricted to those where children live with their father?

Response: We thank the reviewer for proposing these robustness checks. We ran these analyses and placed them in our supporting information (p.11) and in the robustness section (p.28). When we restrict our analyses to children living with their father, our results are largely comparable to our findings based on the full sample and do not change any substantive conclusions.

Here are my suggested changes of wording to replace language that implies causality (not these are suggested changes – happy for the authors to use their own wording, as long as it fixes the issue).

(9) page 6, change "40% of the variation in education can be explained by genetic variation" to "40% of the variation in education is associated with genetic variation", because "explained" implies causality, and there is nothing causal about variance decomposition analysis. Likewise, page 6 "Children with genes that are positively related to higher educational attainment are more open", change to "Children with genes that are positively related to higher educational attainment tend to be more open" or "On average, children with genes.." and then the second part of the sentence reads "..which all result in better educational achievements", which again heavily implies causality and should be changed to sth like "which are linked with better educational achievements".

(10) page 7 "the parent's education PGS not only shapes their own educational attainment" should be sth like "the parent's education PGS is not only associated with their own educational attainment".

(11) page 8 "both fathers' involvement as well children's educational attainment is shaped by the same genetic factors" replace with "both fathers' involvement as well children's

educational attainment is associated with the same genetic factors" ; same page "the same genes that result in higher education, also result in" should say "the same genes that are associated with higher education, are also associated with.."

(12) page 10: "do genes and father involvement independently explain the intergenerational transmission of education" change to sth like "are genes and father involvement independently associated with the intergenerational transmission of education" ; "to what extent do genes explain part of the behaviour mechanism" change to "to what extent do genes account for the behaviour mechanism" or "to what extent do genes confound the behaviour mechanism"

(13) page 11: "we can tap into the causes of the hypothesized correlation " change to "we can test the hypothesized causes of any correlation "

Response: We highly appreciate the detailed suggestions provided by the reviewer to replace language that implies causality. We have changed our language in all abovementioned instances accordingly.

Reviewer #2: The manuscript “The intergenerational transmission of educational attainment: A closer look at the (interrelated) roles of paternal involvement and genetic inheritance” PONE-D-22-08657 investigates the joint effect of paternal involvement and education polygenic score (PGS) in predicting educational attainment, as well as their contribution as mechanisms of intergenerational transmission of education. The study is well motivated (with some reservations described below) to make a reasonable contribution to our understanding in an important topic. However, some empirical decisions and their presentation is confusing and the article overall requires polishing before the publication can be recommended.

Response: We thank the reviewer for their positive evaluation of our manuscript and their constructive comments for improving the manuscript. We address these comments point by point below.

I am admittingly not an expert in path modelling, but I struggle to understand the analysis description:
First, could the authors explain the intuition behind the multilevel models assessing rGE? As described on page 16, they fit model of something like:
Education=education_mother+education_father+PCs+controls+ζ+ε,
and then use ε (individual-level residual, I assume, although the authors do not specify which of the two residual terms ζ/ε, or even both, they use) of the model above to fit:
ε = PGS (or corresponding models examining two dimensions of father’s involvement).
What is the advantage of this approach compared to, for example, the more straightforward method of fitting first a model without mediators and then with mediators, and assessing the attenuation between these models?

Response: We are sorry to hear that the description of our analytical approach wasn’t fully clear. Based on the reviewer’s comment, we have now added to our revised manuscript that we use the individual level error terms (p.21). Our rationale for using multilevel models to assess rGE, and not just simply examine the correlation between the PGS and father involvement, is that these models allow us to examine correlated effects. These correlated effects do not only tell us whether and to what extent there is an association between genes and father involvement, but also whether and to what extent their effects on children’s educational attainment are correlated, which is one of our research aims. Based on the reviewer’s comment, we have now mentioned this advantage of our analytical approach to the revised version of our manuscript (p.21). A final reason for using multilevel models is that we have respondents who are not independent.

Second, for hypothesis 3, why there is no need for other controls that PCs (page 17)?

Response: The reason is that the first 10 genetic PCs are used to control for ancestral differences, but given that siblings already share their ancestry, there is no need to control for the PC in the within-family analyses. We now explain this more clearly in the manuscript (p.21):
To assess the rGE within families, we do not have to control for the first 10 genetic PCs. The reason that we do not have to control for the first 10 PC’s is that siblings share their ancestry, and the genetic PGs are used to control for ancestral differences.

Third, for the description of hypotheses 1, 2 ,4 and 5, it may be beneficial to state explicitly in what way the coefficients are compared. In addition, I would like to see some details on bootstrap simulations (method, has the multilevel structure been taken into account in

sampling, how many replications).

Response: For testing mediation, we do the following (p. 19, methods):

For hypotheses 1 and 2 *These mediated effects (hypothesis 1 and 2) are estimated by multiplying the coefficient of (a) the independent variable on the mediators and (b) the mediators on the outcome (63).*

For hypothesis 4 and 5:
To quantify the extent to which the effect of father involvement in explaining the intergenerational transmission of education is confounded by the education PGS (hypothesis 4), we compare the coefficients of father involvement between the first model (in which only father involvement is included as a mediator) and the third model (in which also the education PGS is included) (64). The other way around, to quantify the extent to which the effect of the education PGS is partly socially confounded (hypothesis 5), and can be explained by father involvement, we compare the coefficient of the education PGS between the second model (in which only the education PGS is included as a confounder), and the third model (in which both the education PGS and father involvement are included).

The bootstrapping was done for the confidence intervals, but as we end up not reporting confidence intervals, we have decided to no longer refer to this procedure in our revised manuscript.

Motivational and interpretational issues:

Authors motivate their focus on paternal involvement based on that “we do expect to see greater variation in paternal than in maternal involvement, and this is our main rationale for choosing to focus on paternal involvement in the current paper” (page 3). Based on Table 1, the difference in standard deviations between both dimensions of paternal and maternal involvement seems to be rather trivial. I tend to think that the paper might be stronger if both paternal and maternal involvement were on the focus, but I do not demand such change if authors think, for example, that this makes focus too scattered. Nevertheless, based on the evidence, I do not buy this specific argument for the current focus.

Response: We thank the reviewer for this observation. We started off this research with this (theoretical) expectation. Our data, however, indeed revealed that maternal and paternal involvement had very similar variation. Although we understand the reviewer’s wish to focus on both mothers’ and fathers’ involvement in the paper, after careful considerations we decided to stay with our original focus, as we fear that the paper will become too large and fragmented. Based on the reviewer’s comment, we did include an observation on the discrepancy between our expectations for differences in variances and the empirical results in the descriptives section of our revised manuscript. This text reads as follows (p. 22):
We expected to find greater variation in father involvement compared to mother involvement. In contrast however, the variation in these two aspects of parental involvement did not substantially differ between fathers and mothers.
In addition, we decided to remove part of the sentence in the intro that mentioned that the greater variation amongst fathers is our main rationale for choosing to focus on paternal involvement. We agree with the reviewer that this argument is not needed to explain to the reader why a focus on fathers’ role in the intergenerational transmission of educational attainment is justified.

On page 24 authors state that “findings from the field of behavioural sciences have likely overestimated the role that fathers’ school-specific involvement plays in the intergenerational transmission of educational attainment.” Is this interpretation consistent with the results? Within-family analysis did not show any attenuations . Although subject of low power and thus only suggestive, wouldn’t this mean that the correlation may stem fully from active rGE. Would this mean that the causal direction would flow from pgs to father involvement, i.e. the involvement is not confounded by the PGS, but acts as one of the mechanisms via which PGS operates. Am I missing something?

Response: We wrote the sentence that “findings from the field of behavioural sciences have likely overestimated the role that fathers’ school-specific involvement plays in the intergenerational transmission of educational attainment.”, based on our finding that genetic confounding is of some importance in explaining the role fathers’ school-specific involvement plays as an underlying mechanism in the intergenerational transmission of educational attainment. This statement is strengthened by the finding that children’s education PGS was a much more important underlying mechanism than the two dimensions of father involvement we considered in the current study.

We are a bit hesitant to add causal interpretations to our findings, also in light of the comments made by reviewer 1. Based on the suggestions of Reviewer 1 and the potential explanation provided here by reviewer 2, we have decided to add the following statement to the discussion section of our revised manuscript. This sentence now reads as follows (p.30): *Also, our suggestive finding of active gene-environment correlation within our sibling sample does not exclude the possibility that genetic confounding is largely caused by child evoked genetic correlation.*

On variables, and related issues:

Does controlling for enrolment in school involve a potential “bad control” problem? It can be a mediator (or even a collider) instead of a confounder given the analysis focus.

Response: We thank the reviewer for this question. Based on it, we ran additional analyses, in which we compared our models with and without including ‘enrolled in education at the last wave’ as a control variable. As can be seen in the table below, there are only very minor differences between these two models. Based on these findings, we have decided to stick to our original models and thus keep the variable ‘enrollment in school’ in our models. Given that we have already reported several robustness checks in our supplementary materials, we would like to propose to only mention these analyses in this revision memo.

Coefficients in model 3 with and without controlling for enrollment:

Coefficient	With enrollment	Without enrollment
School involve	0.050	0.048
Leisure involve	0.031	0.032
pgs	0.219	0.220
Educ father	0.226	0.226
Mediate by school involve	0.007	0.007
Mediate by leisure involve	0.004	0.005
Mediate by PGS	0.065	0.066

On page 15, authors claim to control for “the first 10 principal components (PCs).” PCs of what data? I think I can guess the answer, but scientific writing should not leave readers with guessing games.

Response: We apologize for leaving our readers in the dark. Based on the reviewer’s comment, we have changed this into “the first 10 genetic principal components” throughout the manuscript.

Are there overlapping samples between GWAS and analysis data?

Response: The samples do not overlap. Based on the reviewer’s comment, we have now added this information in the measurement section of our revised manuscript (p. 18).

Could/should the genotyping chip be controlled in the models?

Response: Controlling for the genotyping chip is not needed, as only SNPs that were common across both genotyping platforms were used. Based on the reviewer’s comment, we have now added the information that we use SNPs common across platforms to our revised manuscript (p. 16).

The relative importance of mediators is hard to assess, as they are all in the different scales. Could they be standardized to SD units?

Response: We apologize that this was not clear from the previous version of the manuscript, but we used standardized measures for all of our continuous variables. The coefficients we report are thus standardized beta’s. Based on the reviewer’s comment, we have now added this information in the revised version of the manuscript (result section in the text, underneath figure 3, table 2 and Table S1).

There is a new generation PGS of education (Okbay et al. 2022, Nature genetics, 54(4), 437-449.). Could the new score be accommodated in the revision? However, the improvement is likely to be marginal, as the increase in sample size comes from 23andme, which usually cannot be shared. Thus, if this cannot be easily done, I understand if the authors want to skip this suggestion.

Response: We thank the reviewer for this suggestion. As the reviewer points out him/herself, 23andme cannot be used for the PGS, and will therefore not improve our sample size and also will not lead to substantial improvement. As a consequence, we have not used this new generation PGS of education in the current manuscript.

Reliability assessments of father involvement scales (Cronbach’s α or similar) would be nice.

Response: Based on the reviewer’s suggestion, we added the Cronbach’s alpha to the measurements section of the manuscript. The Cronbachs alphas are 0.77 for father’s school specific involvement and 0.78 for father’s leisure involvement.

Presentational issues:
• Standard errors (or Confidence intervals if authors prefer) could be presented in all tables & Figures. If not possible, then at least in the text when referring to estimates. There is also no

need for vague description of p-values (e.g. “borderline significant” p.21) or asterisks when referring to them in the text. Precise values could be presented as easily.

Response: We added 95% CI in Table 2 in the text. In Figure 5 we added SE. We furthermore removed asterisks and the evaluation “borderline significant” from the text. We also refrain for interpreting findings that were previously labelled as ‘borderline significant’.

• Figures 1-3 could be integrated into different panels of one figure.

Response: We thank the reviewer for this suggestion, and we have created this new figure accordingly.

• I prefer an old-school correlation matrix (with numbers) as Figure 4 relative to heat map.

Response: Based on the reviewer’s comment, we have added a correlation table to our supplementary material. We decided to stick with the heatmap in the manuscript itself, as we feel that this is a much more reader-friendly and concise way of depicting the correlations, but we report the most relevant correlations in the text now.

• Figure 5: It is hard to follow, where 0.016(ns) refers. I guess that between PGS and leisure involvement, but it took its time to understand (if correct).

Response: We regret to hear that Figure 5 was difficult to understand. Based on the reviewer’s comment, we have changed the figure (now labelled Figure 3), we shifted the boxes and lines, in order to make this figure more reader friendly. The reviewer was correct in that the 0.016 refers to the association between PGS and leisure involvement. We hope that the aforementioned changes have made it much easier to interpret this coefficient.

• “Correlated effects” is as exotic term. What is this actually? The correlation, simply, or something else? Possibly clarifying the methods section may help here as well.

Response: We regret to hear that it was unclear what we meant with this term. In our original manuscript, we explained this phenomenon in the first paragraph of the section Analyses-rGE. Based on the reviewer’s comment, we have now rewritten this explanation and placed it in the method section. The text now reads as follows (p.20):

Correlated effects: To examine to what extent the education PGS and father involvement correlate in their relation to educational attainment, we estimated the correlation between children’s years of education predicted from the PGS model and children’s years of education predicted from the father involvement model. To this end, we first regressed children’s years of education on both parents’ years of education, the first 10 genetic PCs, and all other control variables using a multilevel model that takes into account the nested structure of the data. The individual level residuals from this model were used and regressed on the education PGS (model 1), father’s school-specific involvement (model 2), and father’s leisure involvement (model 3). Finally, we assessed the correlation between the predicted values from model 1 with model 2 and model 3, the correlated effects. This approach allows us to not only assess the correlation between the PGS and father involvement, but also whether and to what extent their associations with years of education correlate.

• Overall, it is very unconventional to present outcomes of regression models in the table rows as done in Table 2. This may cause misconceptions.

Response: We thank the reviewer for pointing out that Table 2 needed clarification. In our revised manuscript, we mention that we report Correlation coefficients and β coefficients. The reason we display these regression outcomes in one table with the correlation coefficient is that it is more convenient to compare these estimates in this way.

• There is no need to put different row on "N sibling pairs" in table 2. The old rows, "N individuals" and "N families" could accommodate also sibling design nicely

Response: We thank the reviewer for this suggestion. We have changed our table accordingly.

Issues (again mostly presentational) regarding the analyses of appendix

• Table S1 (upper panel). How can R2 drop between models 2 and 3?

Response: A small drop (from 0.405 to 0.404) in the r2 between the models is possible in a multilevel model, as is explained for example in Snijders & Bosker, 2012 (Snijders, Tom A B, Bosker RJ. How much does the model explain? Multilevel analysis. Sage publications; 2012. pp. 109–119.)

• I would like to see direct effect in the lower panel of table S1

Response: We thank the reviewer for this suggestion. We have added the direct effects in the revised version of Table S1.

.

• Table S2 Contrasting OLS and FE models may be a category mistake. Linear FE models are also typically estimated via OLS. And even if not in this specific case, the essential substance-related difference is not the estimation method.

Response: We agree with the reviewer. The difference is due to the between- versus within-family design. Based on the reviewer's comment, we have now rephrased this in the text, and we have refrained from mentioning FE or OLS.

• P value can never be exactly 0

Response: We agree with the reviewer. The fact that we have p-values that are 0,00 are because of rounding up to two decimals.

Reviewer #3: This study employed the National Longitudinal Study of Adolescent to Adult Health (Add Health) to provide a deeper understanding of the potential role of paternal involvement in intergenerational transmission in academic attainment. The results revealed that both genetic influences as well as father involvement effectively mediate the association between paternal and offspring academic attainment. I believe this study addresses an interesting topic, particularly its emphasis on paternal involvement, is generally well-written, and has the potential to make a meaningful contribution to the extant literature. With that said, however, there are a few areas that can be improved to more effectively display the underlying contribution and further inform future research. I've provided a summary of these areas below with some suggestions for the authors to consider. Best of luck with your revisions and thank you for the opportunity to review your work.

Response: We thank the reviewer for their positive evaluation of our manuscript and their constructive comments. We address these comments point by point below.

On page 2, the authors, rightfully, point out that previous studies have revealed that the association between family environment and educational attainment may be artificially inflated in light of shared genetic influences passed from parents to offspring that collectively contribute to both increased genetic predisposition for variation in educational attainment as well as the environments that parents design for their children. The latter is, at least to some degree, also a reflection of parent predisposition toward educational attainment—and related phenotypes. The resulting covariation between genetic predisposition and these specialized environments that explain variance in educational attainment is, as the authors note, an example of genetic confounding (as well as a passive rGE more specifically). All of this is to say that I completely agree with the authors assessment of this limitation in the literature, but I think it would be beneficial to expand on the underlying meaning of "genetic confounding" in this context as some readers may not be as familiar with this concept and the theoretical and methodological problems that it may give rise to.

Response: We thank the reviewer for this comment and for indicating the need to elaborate on the concept of genetic confounding. Based on the reviewer's suggestion, we have added the following explanation to p. 4 of our revised manuscript:
Genetic confounding arises amongst others because the shared genetic influences parents pass on to their offspring collectively contribute to both increased genetic predisposition for variation in educational attainment as well as the environments that parents design for their children. This might be because traits that are assumed to be environment, such as family SES, are partly explained by genetic factors (7).

Similar to my previous comment, the authors summarize Wertz et al.'s (2020) findings on pages 2-3 of the manuscript. Again, the authors provide a sufficient and accurate description of the concept of "genetic nurture" but I think a slightly more expanded definition and description of the supposed underlying mechanisms underlying genetic nurture would be beneficial for readers that are either unfamiliar with this concept or who are trying to understand how it applies to the current study more directly.

Response. We thank the reviewer for this suggestion. We have added the following explanation underneath our mentioning of the Wertz's paper (p.5):
This, amongst others, shows that part of the association between parental genes and children's education is accounted for by the parenting mothers provide to their children.

I think the authors do a great job of setting up their arguments for shifting focus to paternal involvement within the context of the current study throughout the literature review; however, once they reach the penultimate paragraph (the final full paragraph on page 4) I believe the authors can be a bit more direct. They mention that they are examining paternal involvement and genes (from a GWAS), but they do not provide any indication of how they will examine these two sources of influence. Will the GWAS measure simply serve as a control? Will they examine gene-environment interplay? Again, just a couple of sentences here to flesh things out a bit more may provide readers with valuable information regarding the primary goals of the study.

Response: We thank the reviewer for this suggestion. In line with it, we have added the following sentences at the end of the final paragraph of the introduction (p.7):
We will examine whether and to what extent genes as well as father involvement mediate the relationship between father's and child's educational attainment, whether and to what extent these mediators are correlated, and/or act as important confounders in the intergenerational transmission of fathers' educational attainment.

Hypothesis 1 frame paternal involvement as a mechanism of intergenerational educational attainment. Based on the arguments offered by the authors, I believe this is a reasonable hypothesis. With that said, do the authors believe it is at least possible that at least some of the covariation between paternal educational attainment and involvement is the result of a set of a single suite of genetic influences (or related genetic influences)? I think it is at least possible that educational attainment and parental involvement may be the result of shared genetic influences operating on higher order phenotypes (e.g., impulsivity), of which educational attainment and involvement may reflect more proximately. This could be addressed methodologically with paternal GWAS scores for educational attainment (or involvement, I suppose), but I think the authors need to at least explore/discuss this possibility more directly.

Response: We thank the reviewer for providing us with this alternative hypothesis. Unfortunately, however, we cannot put this hypothesis to the test, as Add Health does not contain paternal genotyping. In the discussion section of our revised manuscript, we now reflect on finding that we were unable to take father's genotype into account. The text reads as follows (p.32):
A suggestion for future research would be to include the genotype of the father, as is done with mother's genotypes in the study by Wertz and colleagues (8). By incorporating father's genotype, one would be able to examine to what extent the relationship between father's education and father's involvement is due to genetic transmission. It would furthermore allow one to examine the extent to which the non-transmitted part of the father's genotype is related to the child's education, and to what extent the association between the non-transmitted part of the genotype and the child's education is accounted for by parental involvement.

The addition of maternal involvement and educational attainment into the multivariate equation significantly strengthens the estimated models. Given the estimated indirect effects, however, it is not currently clear how these measures were "controlled." In other words, did the authors regress the examined outcome (child educational attainment), mediators (father involvement and the PGS), and primary IV (father educational attainment) on the examined controls or just a subset of these measures?

Response: We regret to hear that our analytical approach was not fully clear. We regressed the outcome (child educational attainment) on all controls (age, mother involvement and

education, 10 PCs…). Furthermore, we regressed the first 10 genetic Principal Components on the PGS. We have now added this information in a note to figure 3, as well as in the description of the path model in the Analyses section (p.20).

The extent to which the examined PGS mediated the association between parental and offspring educational attainment was extremely interesting, in my opinion. The authors briefly discuss these findings on pages 22-23, but I think some additional expansion would be useful. More specifically, there has been much discussion surrounding the utility of PGSs as of late with many critics (perhaps, rightfully) challenging the notion of genetic influences as a source of causality. I'm not suggesting the authors tread into these choppy waters, but I do think that framing a PGS as a potential mechanism rather than a source of causal influence may be beneficial given their findings. We are still trying to figure out exactly what the variance explained by a PGS is and how to best leverage these measures. I believe the authors' findings may provide some additional and useful insight in that perhaps we are better suited examining PGSs as a source of intergenerational transmission (when appropriate) rather than a source of more general causality. I don't think the authors need to go too far down this rabbit hole, but some additional expansion here would be beneficial for future research in this area and also highlights an additional contribution of the current study to the extant literature.

Response: We thank the reviewer for these reflections and suggestions. In line with the reviewer's suggestion, we added the following paragraph to the discussion section of our revised manuscript (p.31):
Furthermore, it is important to take into account that PGSs do not imply genetic causality, but are merely based on associations between genetic variants and phenotypes (in our case educational attainment). Therefore, we cannot say that the mediating role of the PGS indicates that this part of intergenerational transmission is due to genes. In fact, this study shows that one of the pathways between PGS and educational attainment is environmental, namely through father involvement.

Reviewer #4: This is an overall well-written paper exploring the mechanisms explaining the intergenerational transmission of educational attainment focusing on paternal involvement. While we know little about how intergenerational transmission works, and know little about maternal influences on educational outcomes, we know even less about paternal involvement in explaining educational outcomes in offspring. The paper adds to this major research gap. The manuscript is methodologically sound and suited for publication in PlosOne. The topic is very important and of interest to researchers from varied disciplines as well as for policymakers and will hopefully spark more research into intergenerational transmission of educational attainment using genetically sensitive designs. I have some minor concerns for the authors to consider.

Response: We thank the reviewer for their positive evaluation of our manuscript and their constructive comments. We address these comments point by point below.

• "To obtain a more complete understanding, the current study integrates insights from the fields of behavioral sciences and genetics and examines the extent to which factors from each field are unique underlying mechanisms, correlate with each other, and/or act as important confounders in the intergenerational transmission of educational attainment." ◊ this to me seemed like the study was looking into several mechanisms, that would be parental, grandparental, sibling, and societal effects on offspring/sibling outcomes, etc. I suggest rephrasing the sentence in the abstract to reflect more precisely what the study was looking into. Paternal effects are grossly understudied, so it is a very valuable study on its own.

Response: We thank the reviewer for their suggestion, and we have adjusted our abstract accordingly, specifying that our focus is on father involvement.

• I suggest adding effect sizes to the abstract. That is, before talking about mediation analyses, state the direct effect, what is the effect size of the correlations between behavioral and genetic influences, etc.

Response: We thank the reviewer for this suggestion and have added the magnitude of the total effect and of the correlations to the abstract.

• Nicely written introduction. I suggest including that SES itself is partly explained by genetic factors, while often assumed to be environmental.

Response: We thank the reviewer for this suggestion. We included the following text in the introduction (p.4):

Genetic confounding arises amongst others because the shared genetic influences parents pass on to their offspring collectively contribute to both increased genetic predisposition for variation in educational attainment as well as the environments that parents design for their children. This might be because traits that are assumed to be environment, such as family SES, are partly explained by genetic factors (7).

It would also be helpful if effect sizes are including in the introduction, for example, the magnitude of correlations between paternal teaching-related activities and offspring educational attainment, etc. (or for example, Children with genes that are positively related to higher educational attainment are more open, agreeable, conscientious, and show more

academic motivation, which all result in better educational achievements – what are the effect sizes here?).

Response: We thank the reviewer for this suggestion. Throughout the theory section we have now included effect sizes, for example:

Previous research found that children who have fathers that are more involved in their schoolwork obtained higher grades in high school (21,29,30) (correlation of 0.113 between father involvement and academic achievement 29). (p. 8)

Children with genes that are positively related to higher educational attainment are more open, agreeable, conscientious, and show more academic motivation (correlations between 0.01 and 0.03), (p. 9)

• A nice addition would have been to include data about parental genotypes- perhaps this could be discussed in the paper?

Response: We agree with the reviewer that it would have been very enlightening to have information on parental genotyping. Unfortunately, as we have also mentioned in response to a comment made by Reviewer 3, Add Health does not contain data on parental genotyping. Based on the reviewer's suggestion, we have now added incorporating parental genotyping as a suggestion for future research in the discussion section of our revised manuscript (p.32).

A suggestion for future research would be to include the genotype of the father, as is done with mother's genotypes in the study by Wertz and colleagues (8). By incorporating father's genotype, on would be able to examine to what extent the relationship between father's education and father's involvement is due to genetic transmission. It would furthermore allow one to examine the extent to which the non-transmitted part of the father's genotype is related to the child's education, and to what extent the association between the non-transmitted part of the genotype and the child's education is accounted for by parental involvement.

• Could you please unpack this? "PGSs cannot distinguish between "direct" genetic effects- associations between genes and education through intelligence and motivation" - What is the direct genetic effect? The genetic variants are not coding for educational outcomes, not even through intelligence and/or motivation. Same here: "and indirect genetic effects -associations between genes and education due to the family environment and parenting practices" Do the authors mean genetic factors explaining family environment and parenting?

Response: We regret to hear that the use of the words 'indirect' and 'direct' genetic effects was unclear. Based on this reviewer's comment and the comment of reviewer 1, we rewrote this paragraph, avoiding the words direct and indirect effects altogether (p. 12):

Yet, these associations do not imply causation, and the pathways from genetic variants to education are diverse [49]. GWAS studies that are used to create PGSs cannot distinguish between associations between genes and education through personal traits, such as intelligence and motivation, and associations between genes and education due to the environment, such as the family environment and parenting practices.

• I suggest discussing the representativeness of the sample. Is the data missing at random? Does the genotyped sample reduce the representativeness? How about information available about paternal involvement? Some sensitivity analysis would be useful.

Response: We thank the reviewer for the suggestion to provide more information on the representativeness of our sample. By including only those respondents for whom their own educational level was available, our sample size decreased from 21317 to 17536 respondents. Based on the reviewer's comment, we checked whether respondents with and without information on their educational attainment (during wave 5) differed in their high school grades (information available during wave 1). Our analyses revealed that respondents for whom we did not have information on their educational attainment during wave 5 had somewhat lower grades in wave 1 compared to those respondents for whom information on educational attainment was available: respectively 14,6% and 10,8 % had a grade D or lower on English in wave 1.

Then, we only selected respondents for whom information on their parents' educational attainment was available. This criterion reduced the sample from 17356 to 14172 respondents. Of the respondents who were dropped from our sample in this step, 47% did not know their father, compared to less than 1% in the sample that remained.

Selecting respondents who provided information on father involvement reduced the sample further down to 13105 respondents. The respondents who were dropped from the sample in this step had somewhat fewer years of education (13.79 years compared to 14.42 years among those who remained in the sample). Those who were dropped from our sample also much more often had a non-resident father (98% compared to 29% among those who remained in the sample).

Finally, removing respondents who were not genotyped reduced our sample further down to 4744 respondents, in which only White respondents remained. There were no substantial differences by educational attainment between those who were dropped from the sample in this step and those who remained in the sample (respectively 14.42 and 14.41 years of education).

We include the following summary of the abovementioned information on attrition in our method section (p. 15-16):

Although Add Health is a nationally representative sample, selecting only respondents with information about their own educational level, the educational level of their parents, and information about parental involvement resulted in a sample that was relatively higher educated and contained respondents who relatively more often lived with both their parents during the first wave of data collection. Furthermore, selecting only genotyped respondents led to a sample that only contained White respondents.

• The methodology is sound, however, I suggest talking about effect sizes rather than significance, e.g., "The effect of the father's school-specific involvement is significantly reduced from 0.056 to 0.050 when including the child's education PGS, which implies 10.7% genetic confounding, while the effect of the father's leisure involvement is not significantly reduced when including children's education PGS.' ◊ this is an interesting but a small effect, it is significant because of the large sample size. It is important to note this.

Response: We thank the reviewer for this suggestion. In the revised version of our manuscript, we removed words like "significant" and "borderline significant" from our text and made sure we report on effect sizes instead. We also refrain from interpreting findings that were 'borderline significant'.

• Was the analysis plan preregistered? How was multiple testing controlled for?

Response: No, we did not pre-register our analysis plan. After careful consideration, we have decided not to correct for multiple testing in our analyses. Our rationale is two-fold. Firstly, our rich theoretical framework yielded only a select number of hypotheses that we tested in our analyses, with only one dependent variable. Thus, the theory-driven nature of our hypotheses counteracted engagement in p-hacking or harking. Secondly, although we understand that, having tested multiple hypotheses, the Reviewer might advise to correct for false positives, the literature does not provide clear directions on whether it was necessary to correct for multiple testing in our study. Our reading of the literature is that it might not always be wise or helpful to correct for multiple testing. Correcting for multiple testing comes with important assumptions that need to be met, such as independence of p-values (for example, with respect to Bonferroni and Bonferroni-Holm corrections). In our paper, recognizing that father involvement and child's PGS are both related to educational attainment, the assumption of independence of p-values is unlikely to hold. Even in the case of less restrictive corrections that learn from the data (for example, Benjamini-Hochberg) we would be challenging our results without being certain about whether such a correction is necessary. In sum, we have given the option of correcting for multiple testing very careful thought. In the end, we have decided not to control for multiple testing and we used regular significant thresholds.

• "Comparing the between-families and within-families associations between father involvement and children's education PGS provides insights into the extent to which the rGE is active or passive." ◊ I do not think you can distinguish between active and evocative rGE?

Response: We agree with the reviewer's assessment, and we have rephrased this sentence as following (p.27):

Comparing the between-families and within-families associations between father involvement and children's education PGS provides insights into the extent to which the rGE is either active/evocative or passive.

In addition, we have also adjusted this wording in other instances in the manuscript.

• I suggest not using the term "borderline significant' it is either significant or not significant. It is especially questionable to interpret the results as showing effect or 'hinting' to an effect.

Response: We agree with the reviewer that we should stay away from using the term 'borderline significant'. Therefore, we have removed this term from the revised version of our manuscript, and we have now refrained from interpreting this finding as significant.

• It is commendable that several robustness checks were done. I suggest doing some checks about missing data and representativeness as well.

Response: As mentioned in response to this reviewer's earlier comment, we have added information on (the checks to assess) representativeness of our data in the Method section of our revised manuscript.

• I suggest adding precise figure legends, what is presented, what are the error terms in parentheses (e.g. figure 5)

Response: We thank the reviewer for this suggestion. We have updated Figure 5, in particular the legends of Figure 5 (which is now labelled 'Figure 3'), and we now display standard errors in parentheses.

---

6. PLOS authors have the option to publish the peer review history of their article (what does this mean?). If published, this will include your full peer review and any attached files.

If you choose "no", your identity will remain anonymous but your review may still be made public.

Do you want your identity to be public for this peer review? For information about this choice, including consent withdrawal, please see our Privacy Policy.
Reviewer #1: No
Reviewer #2: No
Reviewer #3: No
Reviewer #4: No

[NOTE: If reviewer comments were submitted as an attachment file, they will be attached to this email and accessible via the submission site. Please log into your account, locate the manuscript record, and check for the action link "View Attachments". If this link does not appear, there are no attachment files.]

While revising your submission, please upload your figure files to the Preflight Analysis and Conversion Engine (PACE) digital diagnostic tool, https://pacev2.apexcovantage.com/. PACE helps ensure that figures meet PLOS requirements. To use PACE, you must first register as a user. Registration is free. Then, login and navigate to the UPLOAD tab, where you will find detailed instructions on how to use the tool. If you encounter any issues or have any questions when using PACE, please email PLOS at figures@plos.org. Please note that Supporting Information files do not need this step.
